# Case of Caries of the Dorsal Vertebræ, with an Abscess Communicating with the Lungs, and Expectoration of Bone

**Published:** 1841-04-03

**Authors:** John F. White


// MEDICAL EXAMINER.

DEVOTED TO MEDICINE, SURGERY, AND THE COLLATERAL SCIENCES.

No. 14.] PHILADELPHIA, SATURDAY, APRIL 3, 1841. [Vol. IV.

### ORIGINAL COMMUNICATIONS.

*Case of Caries of the Dorsal Vertebræ, with an Abscess communicating with the Lungs, and expectoration of Bone.* By John F. White, M. D.

Before proceeding to the history of this rare and interesting case, I would remark that it was entirely derived from the recollection of the mother of my patient, and recorded as literally as possible.

John Brown, æt. fifteen, was a remarkable healthy and sprightly child until about his fourth year. When, without any obvious cause, he began to trip, and sometimes fall, although no impediment existed in his way; a straw would apparently present a great obstacle, and ofttimes cause him to fall in his efforts to surmount it.

This state of things at first produced no uneasiness in the minds of his friends; but, on the contrary, was attributed to a love of mischief which had previously developed itself in his character, and for this new offspring of which, as on former occasions, the rod was duly administered. Instead, however, of improving under this treatment, the disposition to trip and fall increased rapidly. Day by day he appeared less capable of commanding his lower extremities. A hacking cough manifested itself, and at the expiration of two months he was unable to walk or stand.

He also complained of weakness, and a pain in his right side. No other symptoms recollected.

After having remained in this condition four months, he was enabled, by great exertion, to walk, but not without deformity; for his body was inclined forward, and to the right side, his shoulders were more raised and arms more thrown back than natural, and what attracted particular attention, he was knock-kneed, and walked as if the lower part of his legs did not belong to him.

John had never complained of pain in his back; but this general deformity, and particularly his bent posture, induced his parents to examine it, when "three lumps like knuckles" were observed between the shoulder blades. Up to this period he retained his flesh, but now emaciation commenced, and every day added to his deformity. With great exertion he walked more or less for a month, when he was compelled to give up. The lumps on his back increased rapidly, "and in three months attained apparently their greatest and permanent size, without pain; cough increased, but still dry and hacking. For three or four years he was barely able to crawl. During this time, however, he was attended by one or more physicians, and dosed by his friends with various drugs without apparent benefit. At last a "German doctor" of New York was applied to, who recommended his whole body to be well rubbed with a liniment of his manufacture, of which two or three applications only were necessary to enable him to stand. Then, encouraged by his friends, and making, with timidity, numerous attempts, by the assistance of a hand, chair, &c. he once more acquired the use of his legs. Unfortunately, a month had hardly elapsed before he fell on his back down two or three steps, on account of which, for a year, he was unable even to sit up.

During this time he was again treated with the "German doctor's" liniment, assisted by a good shaking his aunt used liberally to give him three or four times a day, as she had heard it was good for "liver-grown folks."

Since then he has been able to walk, but "soon fatigued and short-winded," often compelled to throw himself forwards across a chair, as the easiest way to obtain relief.

Last fall his cough became more aggravated, still retaining its hacking character, but more frequent, and for the first time as far as can be recollected, attended with expectoration, the character of which, however, was not observed; as the winter advanced, his health declined. His legs gradually lost their power, and were the seat of uneasy and disagreeable sensations, which he could not describe. About the first of the year he expectorated for the first time small pieces of calcareous matter. Cough and expectoration increased, notwithstanding which he continued to attend school until the first of February, when he returned home very much prostrated, from being compelled to stand an hour unsupported, and then roughly handled by a schoolmate.

I was now requested to attend him. On visiting him found him in bed on his knees and elbows, with dyspnœa, and a short, frequent, harassing cough, attended with copious purulent expectoration, with masses of calcareous matter, and one or two pieces like spicula of bone. Hectic fever. On auscultation found every evidence of a cavity at the root of the lung. These symptoms continued with very slight modifications to the day of his death.

About two weeks previous to his death, Dr. Gratz Moses called with me to see him, and on auscultation, and from the expectoration, agreed with me in the opinion that an abscess of the spine, communicating with a cavity in the lungs, existed. Several pieces of bone were voided with the expectoration, the largest about the size of a grain of coffee, and having every appearance of an exfoliation of one of the bodies of the vertebræ. These several pieces were shown to Drs. Horner and Goddard, who pronounced them bone. Before closing the history of this case, I would remark that from the commencement of the disease he experienced difficulty in urinating, and his bowels have always been costive,—indeed, seldom, if ever, acting without the use of medicine.

*Autopsy, March 1st*, 1841, *thirty-six hours after death.*—Present, Drs. Pepper, Wallace, and Moses.

Great emaciation; muscles flabby.

Stomach extremely contracted.

Liver somewhat enlarged, and adherent throughout its superior and posterior surface.

Head of pancreas contained a large, rounded, encysted, calcareous concretion.

Pericardium contained about two ounces of straw-coloured fluid.

Lungs very firmly adherent throughout. Both infiltrated with tuberculous matter, particularly the left, which was almost solidified, and at its summit contained a large anfractuous cavity.

At the roots of both lungs, and communicating with each other, were two cavities about the size of a hen's egg, filled with purulent and calcareous matter and spiculæ of bone. The finger introduced into these cavities passed into a cavity produced by the caries and curvatures of the dorsal vertebra, the bodies of which appeared for the most part to be destroyed, leaving only several loose pieces of spicula of bone. Calcareous matter was also found in the bronchial tubes and glands.

All the dorsal, with one of the lumbar vertebræ, were removed for more minute inspection.

The twelve dorsal were complicated in the curvature, and the entire bodies of the fourth, fifth, sixth, seventh, eighth, ninth, and tenth, were completely destroyed. The eleventh about half gone, leaving the spinal cord on its anterior face exposed, with spicula of bone in contact with it from the third to the tenth dorsal vertebræ inclusive.

